# IGF-IR Targeted Therapy: Past, Present and Future

**DOI:** 10.3389/fendo.2014.00224

**Published:** 2014-12-23

**Authors:** Joseph A. M. J. L. Janssen, Aimee J. Varewijck

**Affiliations:** ^1^Department of Internal Medicine, Division of Endocrinology, Erasmus MC, Rotterdam, Netherlands

**Keywords:** IGF-IR targeted therapy, IGF-IR antibodies, IGF-I receptor, insulin receptor-A, insulin receptor-B, cancer, hyperglycemia

## Abstract

The IGF-I receptor (IGF-IR) has been studied as an anti-cancer target. However, monotherapy trials with IGF-IR targeted antibodies or with IGF-IR specific tyrosine kinase inhibitors have, overall, been very disappointing in the clinical setting. This review discusses potential reasons why IGF-I R targeted therapy fails to inhibit growth of human cancers. It has become clear that intracellular signaling pathways are highly interconnected and complex instead of being linear and simple. One of the most potent candidates for failure of IGF-IR targeted therapy is the insulin receptor isoform A (IR-A). Activation of the IR-A by insulin-like growth factor-II (IGF-II) bypasses the IGF-IR and its inhibition. Another factor may be that anti-cancer treatment may reduce IGF-IR expression. IGF-IR blocking drugs may also induce hyperglycemia and hyperinsulinemia, which may further stimulate cell growth. In addition, circulating IGF-IRs may reduce therapeutic effects of IGF-IR targeted therapy. Nevertheless, it is still possible that the IGF-IR may be a useful adjuvant or secondary target for the treatment of human cancers. Development of functional inhibitors that affect the IGF-IR and IR-A may be necessary to overcome resistance and to make IGF-IR targeted therapy successful. Drugs that modify alternative downstream effects of the IGF-IR, so called “biasing agonists,” should also be considered.

## The Relationship between IGF-I and Cancer

Activation of the insulin-like growth factor-I receptor (IGF-IR) pathway has been found to be essential for initiation and growth of cancers (1). Many types of tumor cells often overexpress IGF-IRs (1). In addition, surrounding stromal tissue of tumor cells produces IGF-I and IGF-II (2) and activation of the IGF-IRs of tumor cells may be mediated by IGFs in a paracrine and autocrine way (3). Prospective studies suggest that individuals with circulating IGF-I concentrations at the high end of the normal range have an increased risk for several common cancers (1, 4). In many tumors, binding of IGF-I and IGF-II to the IGF-IR, inhibits apoptosis and promotes cell proliferation (5). In the last years, insulin has been also associated with the development of cancer in subjects with type 2 diabetes. Although all the underlying mechanisms need to be clarified, it has been suggested that hyperinsulinemia upregulates growth hormone receptor (GHR) expression and thereby upregulates hepatic IGF-I production (6). In addition, hyperinsulinemia also suppresses the levels of IGFBP1 and IGFBP2, thereby increasing IGF-I bioavailability (7).

## The Complexity of the Insulin-IGF Family

The insulin-IGF family comprises a complex molecular signaling pathway (8). Depending on which regions are being compared, the IR and IGF-IR have sequence similarities varying from 41 to 84% (9). Both the IR and IGF-IR belong to the family of ligand-activated receptor kinases. The gene coding for the IR is localized on chromosome 19 and is composed of 22 exons (10, 11). Alternative mRNA splicing results in the expression of two isoforms: one containing exon 11 (the “classical” IR-B) and one missing exon 11 (the IR-A) (12). Exon 11 encodes for 12 amino acids localized at the C-terminal part of the alpha chain of the IR. Deletion of this exon has important functional consequences: IR-A has high affinity for insulin and IGF-II but binds IGF-I with low affinity. IR-A binds insulin with 1.5-fold higher affinity than IR-B and possesses a higher dissociation and internalization rate (13). Therefore, in cells with increased IR-A: IR-B ratios, insulin mainly signals through IR-A (14). IR-B is responsible for the classic metabolic responses induced by insulin, and also binds IGF-I and IGF-II with low and intermediate affinity, respectively (15). The expression profile of both IR isoforms is tissue-specific (12, 16). Interestingly, an increase in the IR-A to IR-B ratio have been reported in type 2 diabetes (17, 18).

All this information is mainly obtained by measurements of mRNA levels of IR-A and IR-B in different tissues. However, at present there is no good information about the exact expression of protein levels of IR-A and IR-B at the cell surface due to a lack of antibodies which can distinguish both isoforms of the IR.

In contrast to insulin, most of the IGFs in the circulation and in other extracellular fluids, form complexes to six high-affinity IGF-binding proteins (IGFBPs) (5). IGFBPs regulate the half-life and bioavailability of IGF-I and IGF-II and modulate their accessibility to the receptor (19). Under physiological circumstances but also in cancers partial proteolysis of IGFBP-3 and other IGFBPs may be an important mechanism for regulating IGF-I bioavailability (20).

## The Role of IGF-Insulin Hybrid Receptors and Their Cross-Talk with Other Receptors

**Figure 1:**
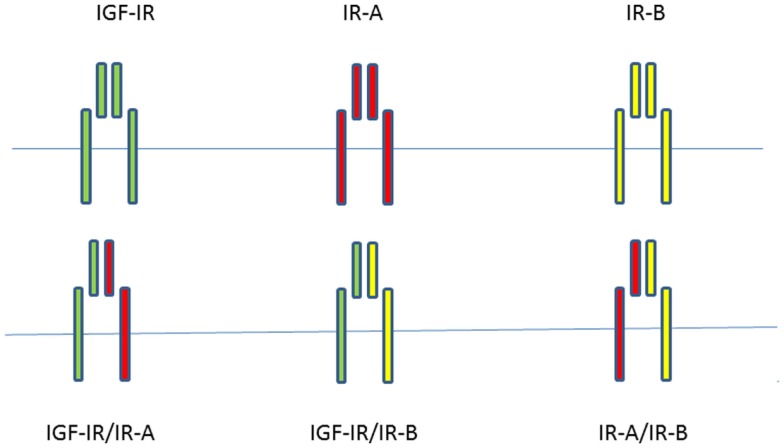
**When considering all possible combinations of homodimer and hybrid receptors of the insulin/IGF signaling pathway, there are at least six potentially tyrosine kinase receptors involved in signal transduction**.

Since most mammalian cells express both the IGF-IR and the IR, although at different expression levels, functional heterodimers can form between the IGF-IR and IR isoforms (15). A significant fraction of both IRs and IGF-IRs is present as hybrids in most mammalian tissues, including those that are considered to be classic targets of insulin (21). The IR-A/IGF-IR hybrids bind insulin and both IGFs with similar affinity, whereas IR-B/IGF-IR hybrids mainly behave like an IGF-IR, with high affinity for IGF-I, low affinity for IGF-II, and insignificant affinity for insulin (22). Slaaby et al. postulated that a possible explanation for this latter phenomenon may be that to achieve high-affinity binding, IGF-I requires a receptor monomer, while insulin requires a homodimeric IR (23). Thus, IGF-I may bind to the IGF-IR half of a hybrid receptor irrespective of what the molecular structure of the other half receptor is, while insulin requires a homodimeric IR to achieve this (23). Both IGFs and insulin stimulated cell proliferation and migration more effectively in cells containing IGF-IR/IR-A hybrids than in cells containing IGF-IR/IR-B hybrids (22). When considering all possible combinations of homodimer and hybrid receptors of the insulin/IGF signaling pathway, there are at least six tyrosine kinase receptors potentially involved in signal transduction (Figure 1).

The IR/IGF-IR signaling pathway also has extensive cross-talk with the GHR, estrogen receptor (ER), androgen receptor (AR), epidermal growth factor receptor (EGFR), and human epidermal growth factor receptor 2 (HER-2) signaling pathways (24–27) (see below). On the other hand EGFR mediated growth requires the presence of functional IGF-IRs for its mitogenic activities (28).

While IGF-IR can heterodimerize with the IRs, as discussed above, it can also form heterodimers with receptors from other families (8). For example, the IGF-IR may form a heterodimer with the EGFR (29). Inhibition of one receptor in these hybrids may shift the signaling pathway in favor of the other available counterpart receptor (8, 30, 31).

## Strategies to Target the IGF-IR in Cancer

Specific signaling pathway (like IGF-IR pathway) dependence of certain cancers creates, at least theoretically, an Achilles heel for tumor maintenance, which can be exploited therapeutically (32). As an anti-cancer target, the IGF-IR has been studied in many clinical trials over the past years (33). Three major strategies have been used: (1) monoclonal antibodies against the IGF-IR, (2) monoclonal antibodies against IGF-I or IGF-II, and (3) tyrosine kinase inhibitors of the IGF-IR (33). Monoclonal IGF-IR antibodies block ligand binding and induce receptor internalization and degradation (34). As a class effect, IGF-IR blocking drugs cause insulin resistance, hyperinsulinemia, and (often mild and reversible) hyperglycemia (35). Besides through decreased IGF-I action, insulin resistance induced by IGF-IR blocking drugs is also thought to be caused by an increased secretion of GH and some IGFBPs (36). The increased GH secretion is the result of a direct blockade of the pituitary IGF-IRs by these agents (36). Monoclonal antibodies against IGF-I or IGF-II act by neutralizing IGF-I and IGF-II, preventing receptor activation without affecting glucose tolerance (37). The majority of IGF-IR tyrosine kinase inhibitors act by competing with ATP for binding in the IGF-IR kinase domain, although also non-competitive agents are being developed (37). They may induce hyperglycemia by direct inhibition of IR-kinase (37).

Although, initially promising effects have been reported in phase 2 trials, more extensive experience has learned that a potential clinical benefit for monotherapies blocking IGF-I or the IGF-IR is only limited to a small subset of patients with specific cancers (sarcoma, Ewing sarcoma non-small cell lung cancer and some other chemotherapy-refractory solid tumors) (38–40), while the majority of studies have been disappointing and have failed to show any clinical benefit in the treatment of cancers (33). In the next sections, we will discuss some background and possible reasons for the failure of these therapies.

## The Feasibility of the IGF-IR as Anti-Cancer Target

Recent DNA and RNA sequencing projects have revealed recurrent somatic alterations in several genes that are drivers of oncogenesis. Most cancers contain many mutations and are thus not dependent on one single oncogenic mutation (32). They activate RAS, BRAF, EGFR, and many others. These driver alterations can give rise to a tumor dependency on a particular signaling pathway (32). However, in clinical settings no cancer specific mutations of the IGF-IR or its ligands have been described to date (41). Moreover, the IGF-IRs of cancer cells also do not contain intrinsic (post) receptor abnormalities (42).

Owing to the presence of an intracellular tyrosine kinase domain, the IGF-IR is usually classified as a tyrosine kinase receptor. Accordingly, phosphorylation was until very recently, considered to be the central process governing IGF-IR signaling (42). As a consequence, to date most therapies targeting the IGF-IR have mainly been designed aiming to block phosphorylation mediated signaling by preventing receptor–ligand interaction or by limiting kinase activation (42).

## Consequences of IGF-IR Targeted Therapy in Cancer

The common mechanisms of action of IGF-IR targeted antibodies are that they block the IGF-IR from ligand binding and induce internalization/degradation of the IGF-IR (43). Most developed antibodies directed against the IGF-IR are highly specific for the IGF-IR and do not bind to IRs. Nevertheless, it has been found that antibodies directed against the IGF-IR may not only downregulate IGF-IR homodimers but also the IGF-IR/IR hybrids (44). Interestingly, in a model of prostate cancer, following IGF-IR inhibition, the IR was able to compensate for and mediate IGF-I induced mitogenic signaling (45). Moreover, in breast cancer cells, downregulation of IGF-IRs by siRNA sensitized these cells to insulin-mediated activation of intracellular downstream signaling pathways (46).

## Mechanisms Responsible for Failure of IGF-IR Directed Therapy

The following reasons for the failure of IGF-IR targeted therapy to inhibit the growth of human cancers have been suggested (47):

(1) Mutations in PI3K (or PTEN deletions) constitutively activate Akt (and subsequent cell cycle progression genes) and render cells resistant to IGF-IR targeted therapy. This has not been proven so far.
(2) Targeting the IGF-IR in some cells (e.g., hematopoietic precursors and neuronal cells) could actually inhibit a tendency to differentiation.
(3) An increased IR-A cell surface expression upon downregulation of IGF-IR signaling. The Vignieri–Belfiori group has convincingly demonstrated that stimulation of the IR-A – especially by IGF-II – may induce mitogenic signals and replace IGF-IR signaling in human tumors (13, 47). Thus activation of the IR-A by IGF-II bypasses IGF-IR signaling and its inhibition.
(4) Anti-cancer treatment may cause a reduction of IGF-IR expression at the cellular membrane. For example, treatment of human breast tumors with the anti-estrogen tamoxifen causes reduction in membranous IGF-IR expression (48). A reduction in membranous IGF-IR expression may occur as an adaptive resistance mechanism and this may play an important role in the inability to sustain IGF-IR blockade (8). In addition, absence of *N*-linked glycosylation has been reported to change insertion of the IGF-IR into the cell membrane and induces resistance to IGF-IR directed antibodies (49). Nuclear translocation of the IGF-IR may be another factor, which limits accessibility and actions of IGF-IR directed antibodies. On the other hand, exclusive nuclear localization of IGF-IR has been reported to be associated with a better response when patients were treated with IGF-IR targeted antibodies (50).
(5) Most important is the complexity of regulatory mechanisms in cancer cells, including the existence of subpopulations in each cancer with different mutations and sensitivities. As discussed in the previous paragraph, the IGF system is not a unique tumor driver and each of these subpopulations can be regulated by a variety of genes and large and small non-coding RNAs (the “dark matter” of cellular function) (47). Moreover, signaling pathways in cells are interconnected and these interactions are dynamic in time (51). It has become clear that all pathways previously thought to be linear are in fact highly interconnected into complex signaling pathways (32). Receptor cross-talk can occur through ligand interactions or via common downstream molecular pathways and modulation (8). This cross-talk gives tumors additional signaling opportunities and the potential to evade regulatory checkpoints (8). For example, the inhibition of the IGF-1R pathway by cixutumumab results in stimulation of the Akt/mTOR pathway by increasing synthesis of EGFR, Akt1, and antiapoptotic surviving proteins (52).

As discussed above, IGF-IR blocking drugs may also induce hyperglycemia, hyperinsulinemia, and an increased GH secretion. This may paradoxically contribute to proliferation of tumor cells by several mechanisms:

(1) Although phosphorylation of IGF-IR tyrosine residues is generally considered to be the initial activation step within the intracellular IGF-IR signaling pathway, it has been found that cells undergo a signaling switch under hyperglycemic conditions (53). After this switch, a completely different mechanism is utilized to activate mitogenic pathways downstream of the IGF-IR and this activation is independent of IGF-IR tyrosine phosphorylation (54).
(2) Secretion of insulin is mainly regulated by glucose levels. IGF-IR blocking drugs increase glucose levels, which may induce hyperinsulinemia and thereby tumor growth by directly stimulating IR-A and indirectly by inducing resistance to chemotherapy (55).
(3) An increased expression of the GHR is a characteristic of a variety of human cancers while GH may induce tumor growth, independent of IGF-I action (56).

**Figure 2:**
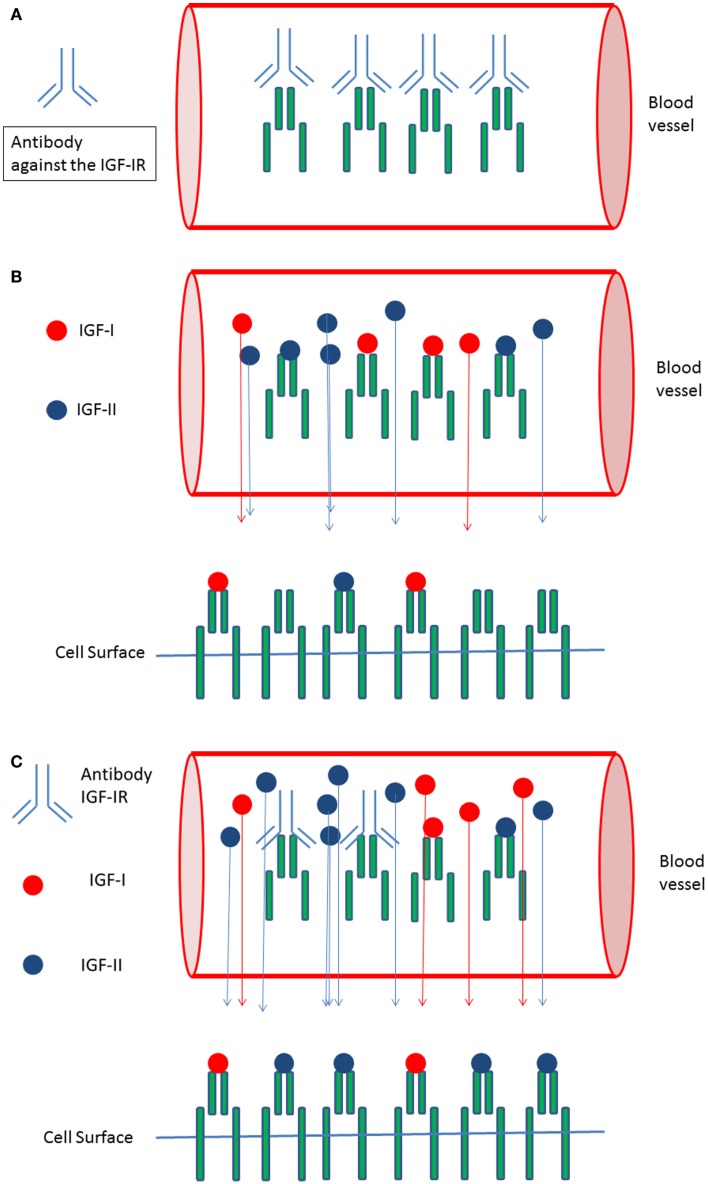
**Circulating IGF-I receptors in cancer patients may form complexes with IGF-IR directed antibodies and this may reduce therapeutic effects of IGF-I receptor antibodies *in vivo*: (A)** In the circulation soluble IGF-I receptors and antibodies directed against the IGF-IR may form complexes. This may limit the availability of IGF-IR antibodies leaving the circulation and reduce the amount of IGF-IR antibodies leaving the circulation to inhibit IGF-I receptors present at the cell surface of cancer cells. **(B)** Only “free” IGF-I and IGF-II may leave the circulation to bind to IGF-I receptors present at the cell surface of cancer cells. Complexes formed between the IGFs and the soluble IGF-Rs are to big to pass the vessel wall and to leave the circulation. **(C)** Formation of complexes between IGF-IR directed antibodies and soluble IGF-IR displace IGF-I and IGF-II from the soluble IGF-I receptors thereby (paradoxically) increasing the total amount of free IGF-I and IGF-II that can leave the circulation to stimulate IGF receptors displayed at the cell surface of cancer cells.

Recently circulating IGF-IR levels were reported (57). We hypothesize that circulating (soluble) IGF-IRs in cancer patients may be another important factor responsible for the observed discrepancy between *in vitro* and *in vivo* effects of IGF-IR targeted therapy: antibodies against a tumor-target largely remain in the blood and usually no more than 20% of the administered antibody dose typically interacts with the tumor (58). Formation of complexes between the soluble IGF-IRs and antibodies directed against the IGF-IR may further reduce the amount of IGF-IR antibodies leaving the circulation to interact with IGF-IRs expressed on the surface of cancer cells (Figure 2A). In addition, these complexes displace IGF-I and IGF-II from the circulating soluble IGF-IRs thereby (paradoxically) increasing the amount of free IGF-I and IGF-II that can leave the circulation to stimulate IGF-IRs displayed on the surface of cancer cells (Figures 2B,C).

## Is There Still a Potential for IGF-IR Targeted Therapy in Cancer?

Given the complex signaling pathways that converge on those activated by the IGF-IR, it is not surprising that for most cancers IGF-IR targeted monotherapy *in vivo* has been disappointing (8, 59). Although IGF-IR targeted monotherapy has essentially been abandoned, it is still possible that targeting the IGF-IR may have an important role as adjuvant treatment of human cancers (47) (see below).

Since the IGF-IR has extensive cross-talk with other receptor tyrosine kinases and their downstream effectors, inhibition of the IGF-IR by a specific antibody may be compensated by other pathways (37). The most obvious candidates responsible for this compensation are the IR-A and IGF-II. *In vitro*, we recently showed that circulating IGF-II may contribute substantially to IR-A and IR-B signaling (60). It has been also suggested that therapeutic interventions of cancers may trigger a phenotype switch in surviving cells to a more primitive and immature cell state (61). In favor of this latter possibility, as discussed above, the IR-A and IGF-II are frequently highly expressed by human tumors. In addition, there is further evidence that resistance to IGF-IR directed therapy is the direct result from formation of IGF-I/IR-A hybrids and IGF-II signaling via the IR-A isoform (37). Thus the development of functional inhibitors that affect IGF-IR and IR-A may be necessary to overcome resistance to IGF-IR directed therapy.

On the other hand, IGF-IR blocking therapy is expected to be most effective in tumors with increased IGF-IR and poor IR-activation. Thus, identification of a subset of patients most likely to benefit from IGF signaling interference should be pursued.

Combining the diagnostic and therapeutic potential of an antibody, thereby selecting those patients who are most likely to benefit from antibody treatment, could be an important step to improve IGF-IR targeted therapy (62). In contrast to immunohistochemical imaging of the IGF-IR, molecular IGF-IR imaging (for example ^111^In-R1507 SPECT) *in vivo* might be able to predict and to identify who will benefit from IGF-IR targeted therapy (63).

Combining IGF-IR targeted therapy to chemotherapy may be another potential successful strategy since IGF-I may protect tumor cells from being killed by cytotoxic drugs (37, 64). In addition, this may help to suppress chemotherapy induced IGF-IR activation and DNA repair mechanisms (37). Furthermore, the feasibility and timing of combining multiple targeted therapies (IGF-IR and IR-A) and conventional cytotoxic drugs need to be explored. Recently it was suggested that the IGF-IR also behaves like a functional receptor tyrosine kinase/G-protein related coupled receptor (GPCR) hybrid “borrowing” components of GPCR signaling (42). As a consequence, the IGF-IR (and IR) can activate signaling as a GPCR, using different G-proteins (42). IGF-I activity and its biological effects are further controlled by a variety of adaptor proteins/signaling proteins through IGF-IR posttranslational modifications including tyrosine and serine phosphorylation, dephosphorylation, ubiquitination, and sumoylation (42). Therefore potential drugs that modify alternative downstream effects of the IGF-IR, the “biasing agonists,” should also be considered (42).

## Author Contributions

Both Joseph A. M. J. L. Janssen and Aimee J. Varewijck researched data, wrote manuscript, and reviewed/edited the manuscript.

## Conflict of Interest Statement

The authors declare that the research was conducted in the absence of any commercial or financial relationships that could be construed as a potential conflict of interest.
